# Intravenous thrombolysis in ischemic stroke patients based on non-contrast CT in the extended time-window

**DOI:** 10.3389/fstro.2022.1026138

**Published:** 2022-11-23

**Authors:** Julia Emde, Romy Baumgart, Niklas Langguth, Martin Juenemann, Stefan T. Gerner

**Affiliations:** Department of Neurology, University Hospital Giessen-Marburg Location Giessen, Giessen, Germany

**Keywords:** ischemic stroke, intravenous thrombolysis (IVT), imaging, extended time-window, advanced imaging, Alberta Stroke Program Early CT Score (ASPECTS), non-contrast CT

## Abstract

**Purpose of review:**

Recent trials provided evidence for safety and efficacy of intravenous thrombolytic therapy (IVT) in ischemic stroke patients beyond the 4.5 h time-window if ischemic penumbra is present in multimodal imaging. However, advanced imaging by either Magnet Resonance Imaging (MRI) or Computed Tomography Perfusion (CTP) is not available 24/7 at most stroke-centers. Therefore, the current review addresses the use of non-contrast CT (NCCT) to identify ischemic stroke patients suitable for IVT in the unknown or extended time-window in terms of efficacy and safety.

**Recent findings:**

The current data on NCCT based IVT strategies in ischemic stroke patients presenting in the unknown or late time-window are relatively scarce and mainly provided by small retrospective samples. One larger registry (TRUST-CT) underlines the safety and efficacy of IVT without advanced imaging with more IVT-patients reaching an excellent outcome compared to the non-IVT treated control group. Current meta-analysis provides evidence that the rate of symptomatic intracerebral hemorrhage (sICH) is similar in the wake-up and unknown onset time-window compared to the 4.5 h time-window if patients are selected by NCCT. Results of the upcoming TWIST-trial investigating Tenecteplase (TNK) for NCCT-based IVT revealed no signals regarding an increased rate of sICH, however there was no benefit regarding functional outcomes.

**Summary:**

So far, it is not well-established whether advanced imaging is indispensable and NCCT could be sufficient to identify stroke patients in the extended window who would benefit from IVT-treatment. However, current data suggests the safety of NCCT-based IVT in the extended time-window. Therefore, unavailable advanced neuroimaging should not cause delay, or even exclusion of patients from IVT and other recanalizing therapies *per se*.

## Introduction

Intravenous thrombolysis (IVT) with recombinant tissue plasminogen activator (rt-PA) given within 4.5 h of onset improves clinical outcomes in patients with ischemic stroke (Berge et al., 2021). However, only a minority of patients, 20–25%, enter the hospital within this established timeframe for treatment (Adeoye et al., 2011). Additionally, the time of onset is unknown in up to 20–25% of patients (Thomalla et al., 2020).

IVT is time-dependent with early treatment resulting in increased odds of a better outcome illustrating the consequences of any delays (e.g., odds ratio 1.75 for IVT up to 3.0 h and odds ratio 1.25 for IVT up to 4.5 h). A treatment effect was also shown beyond the limit of 4.5 h up to 6.3 h in a regression model indicating that for some patients IVT is potentially beneficial in an extended time-window (Emberson et al., 2014).

Several studies have already investigated IVT in the extended time-window or if the onset of stroke is unknown. Previous trials, such as the WAKE-UP, ECASS-IV, EXTEND, and EPITHET trials as well as meta-analyses of these data underline the efficacy of IVT in selected patients with either clinical or imaging-based penumbral-ischemic core mismatch. These advanced imaging methods rely on perfusion-diffusion MRI, mismatch between MRI diffusion weighted imaging and fluid attenuated imaging (DWI-FLAIR-mismatch as used in WAKE-UP trial), or CT Perfusion (CTP) (Davis et al., 2008; Thomalla et al., 2018, 2020; Campbell et al., 2019; Ringleb et al., 2019; Ma et al., 2022). Although the efficacy and safety of IVT has been shown for those patients who fulfilled these relatively strict imaging criteria, it remains unclear whether there may be a benefit for patients not fulfilling these imaging criteria as well. Moreover, previous studies suggested in patients with stroke onset <3 h that DWI-FLAIR-mismatch can be absent in up to 40% of the cases, a finding that may lead to exclusion of suitable wake-up stroke patients from thrombolytic therapy (Thomalla et al., 2011). Another shortcoming is known for CT-perfusion, in which infarct core overestimation seems to be common resulting in unnecessary denying of IVT-treatment to patients (Garcia-Tornel et al., 2021).

The aforementioned advanced imaging techniques are more time consuming and can potentially lead to treatment delays in the very time sensitive setting of acute stroke care (Sheth et al., 2013). Further, CT-based advanced imaging implies higher radiation exposure and contrast load to the patient. It has to be kept in mind that 24/7 availability of these advanced imaging modalities is expensive and cannot be ensured by every hospital (Kane et al., 2008). Furthermore, perfusion-imaging has known shortcomings to detect small hypoperfused areas, in particular lacunar ischemic lesions and brainstem ischemia. But should patients be excluded of potentially effective treatment options because of the absence of multimodal imaging?

All this leads to the question whether findings on non-contrast CT (NCCT) are adequate to make a decision on IVT treatment. Hence, this review first looks from a radiological point of view at merits and limits of the ASPECT Score derived from NCCT and whether it correlates with parameters provided by multimodal imaging like infarct core volume. Second, it addresses the question of whether IVT is effective and safe in NCCT-selected patients up to current knowledge.

## Non-contrast CT

### Radiologic features

NCCT is highly sensitive to rule out intracranial hemorrhage which represents the major contraindication for IVT treatment in patients with stroke-symptoms (Powers et al., 2018). Up to 60% of ischemic stroke patients show pathologic changes in the initial NCCT scan if performed during the first 6 h after onset. One specific sign is the hyperdense artery sign suggesting occlusion of a proximal artery. Hypoattenuation on the NCCT-scan of stroke patients is highly specific for irreversible brain damage due to ischemia. At the same time, the absence of hypoattenuation is associated with better clinical outcomes (Von Kummer et al., 2001). Moreover, hypoattenuated regions are suggested to represent the infarct core, whereas regions that are isodense and swollen rather have increased cerebral blood volume and are more likely to indicate penumbral areas. Although non-contrast CT is not sensitive for detection of infarct core and penumbra, information on tissue viability can be obtained because of its specific appearance (Muir et al., 2007). Early radiologic changes during the first 3 h after stroke onset comprise a reduced differentiation between white and gray matter (e.g., insular ribbon sign or disappearing basal ganglia sign) and parenchymal swelling with a decrease in the size of ventricles and/or sulci. Patients where more than one third of the middle cerebral artery (MCA) territory is affected are more likely to develop a malignant infarction with severe brain swelling. In a subacute state of stroke, the infarction appears increasingly demarcated and hypoattenuated. Further, swelling can increase, and hemorrhagic transformation can occur from two to seven days after stroke.

To grade the extension and severity of strokes and to identify the early and potentially reversible radiologic signs several scores have been developed. The most established one is the Alberta Stroke Program Early CT Score (ASPECTS) as a 10-point-scale of cerebral NCCT scans to evaluate MCA strokes. In two standardized axial CT cuts, 10 regions of the MCA territory were determined. One at the level of the thalamus and basal ganglion and the other from the cranial to the ganglionic structures. A normal CT scan receives 10 points. For every area with early ischemic changes, implying parenchymal hypoattenuation relative to contralateral brain structures or focal swelling in terms of any focal narrowing of the cerebrospinal fluid, one point is subtracted from 10 (Pexman et al., 2001). In general, ASPECTS has shown an association between higher values and greater benefit from intravenous thrombolysis (e.g., in the NINDS trial). There is an inverse correlation between ASPECTS and functional outcomes (Schroder and Thomalla, 2016) and between ASPECTS and the National Institutes of Health Stroke Scale (NIHSS), respectively. Moreover, an ASPECTS of 7 or lower has a high sensitivity for prediction of symptomatic intracerebral hemorrhage (odds-ratio ~14) (Barber et al., 2000).

The ASPECTS seems comparable to perfusion parameters like CTP ischemic core and DWI lesion volumes regarding the prediction of clinical outcomes (Haussen et al., 2016; Nagel et al., 2020) and shows moderate correlation compared to MRI diffusion-weighted sequences and CT-perfusion for estimation of the ischemic core volume (Bal et al., 2015; Nagel et al., 2020; Voleti et al., 2021). These correlations between ASPECTS and multimodal imaging increase with time, especially in late presenting stroke patients (Bal et al., 2015), those with large infarct core volumes (Demeestere et al., 2017; Nagel et al., 2020) or large vessel occlusion (Desai et al., 2020; Nannoni et al., 2021). Usage of automatically derived acute ischemic volumes from e-ASPECTS seems to further improve these correlations. Another advantage using e-ASPECTS software is that interrater variability in the interpretation of ASPECTS can be partly overcome (Nagel et al., 2020).

## IVT for ischemic stroke in the unknown and late time-window

Even if current data on NCCT based IVT strategies in ischemic stroke patients presenting in the unknown or late time-window are relatively scarce, there are some investigations analyzing outcomes especially in wake-up patients (for study details see Table 1).

**Table 1:** Studies investigating safety of NCCT based IVT in SUO and WUS patients.

**Study**	**Date**	**Design**	sICH rate^*^
TRUST CT	2020	Pre-defined retrospective analysis of a dedicated, prospective, multicenter, international registry	3.4% IVT patients vs. 0.9% controls (aOR 7.9, 95% CI 0.65–96, *P* = 0.1)
Zha et al. (2022)	2021	Retrospective, single center	1% IVT patients vs. 4% controls (OR 0.3; 95% CI 0.0–1.9)
Zha et al. (2022)	2021	Meta-analyses of three studies	2.1% IVT patients vs. 3.4% controls (OR 1.01; 95% CI 0.45–2.28)
Armon et al. (2019)	2019	Retrospective, single center	SUO 1.8% and WUS 3.7% No control group
TWIST	2021	Multi-center, prospective, randomized-controlled	3.1% IVT vs. 1.0% controls (*P* = 0.09)

aOR, adjusted odds ratio; OR, odds ratio; CI, confidence interval; IVT, intravenous thrombolysis; SUO, stroke of unknown onset; WUS, wake-up stroke;

* sICH is not defined uniformly over mentioned studies.

TRUST-CT (Thrombolysis in Stroke with Unknown Onset Based on Non-Contrast Computerized Tomography), one larger international multicenter registry-based study included 117 patients treated with non-contrast computed tomography-based IVT and 112 non-treated controls (Sykora et al., 2020). IVT was applied if NIHSS was ≥4 and initial ASPECTS was ≥7. 33.3% of IVT-treated patients reached excellent outcomes in terms of a modified Rankin scale (mRS) of 0 or 1 compared to 20.5% in the control group (adjusted *p* = 0.05). An improvement in the NIHSS score of ≥4 NIHSS points was observed in 48.8% of thrombolysis patients vs. 23.2% in the control group (adjusted *p* < 0.001). The rate of symptomatic intracranial hemorrhage (sICH) was not significantly higher in the IVT group (four patients vs. one control patient). The maximum time from last seen well and admission was ~20 h for thrombolysis patients. These findings indicate the feasibility, efficacy, and safety of NCCT based thrombolysis in wake-up strokes and strokes of unknown onset (WUS/SUO) (Sykora et al., 2020).

Zha et al. (2022), conducted a retrospective single center study and a meta-analysis regarding IVT in patients with wake-up stroke/stroke of unknown onset based on NCCT which underlines the TRUST-CT results. A total of 1,827 thrombolysis treated stroke patients were analyzed in consideration of appearance of sICH and 90-days-mRS comparing WUS/SUO patients (*n* = 93), and patients in the 4.5 h time-window (*n* = 1,734). Outcomes were similar in both groups. They found no statistical difference in the rate of sICH (1% WUS/SUO vs. 4% controls), both in their own retrospective cohort and in three other studies. The pooled meta-analysis of 485 WUS/SUO patients from seven studies revealed a 2.1% incidence of sICH (Zha et al., 2022).

In a similar smaller retrospective chart review with 306 IVT patients included, Armon et al. (2019), confirmed the safety of NCCT-based thrombolysis based on low sICH rates, both for SUO (1.8%) and for WUS (3.7%). Patients only got IVT treatment providing a normal CT scan (ASPECTS = 10), so that a safe IVT treatment with a 6 h time-window for those patients was proposed by the authors. The time of last seen well was not documented and a comparison with a control group was not performed.

The TWIST-trial (Tenecteplase in wake-up ischemic stroke) is a multi-center, prospective, randomized-controlled trial investigating treatment with Tenecteplase (TNK) (*n* = 288) vs. standard care (*n* = 290) in patients who wake up with acute ischemic stroke symptoms and can be treated within 4.5 h upon awakening based on NCCT (Roaldsen et al., 2021). First results were presented at the European Stroke Organization Conference, 2022 in Lyon. So far, it revealed no signals regarding an increased rate of sICH, however there was no benefit in the ordinal-shift analysis of the mRS-score among the TNK and placebo-group.

As it is likely that onset of ischemia in WUS happens in the early morning hours near to awakening, affected patients probably are within a 4.5 h time-window (Thomalla et al., 2018). This is underlined by retrospective analysis which suggested a similar rate of ASPECTS between 8 and 10 in patients presenting in the unknown time-window compared to those presenting within 4 h of symptom onset (Huisa et al., 2010).

Only the Third International Stroke Trial (IST-3) included patients in a known but late time-window based on NCCT. In this international, multicenter, randomized, open-treatment trial 3,035 patients presenting within 6 h after symptom onset were allocated into an IVT-treatment group or into the control group. Half of included patients were older than 80 years. At 6 months, 37% of patients in the rt-PA group vs. 35% in the control group were independent, which was defined by an Oxford Handicap Score of 0–2. An ordinal analysis showed an overall significant shift in these scores (OR 1.27, *p* = 0.001) in favor of rt-PA treatment. Fatal or non-fatal symptomatic intracranial hemorrhage within 7 days occurred in 7% in the IVT-group vs. 1% in the control group (adjusted OR 6.94) and deaths after 6 months were equal in both groups (27 %). Subanalysis revealed 240 of 507 (47.3%) patients treated with rt-PA vs. 213 of 500 (42.6%) patients treated with placebo within the 4.5–6 h timeframe to achieve an independent outcome at 90 days resulting in an adjusted odds-ratio of 1.31 (0.89–1.93) in favor of IVT (IST-3 Collaborative Group et al., 2012). However, these sub-results should be interpreted cautiously as this trial is highly likely prone to bias, especially for patients presenting within the 4.5–6 h time-window due to shortcomings in its trial design (Lyden, 2012).

As the remaining mentioned data deals with WUS/SUO patients, apart from the IST-3 Trial, there is a lack of current research concerning patients in a known but late time-window apart from 4.5 h of onset due to the already established algorithm using advanced imaging to identify patients eligible for IVT. As a consequence, the quoted results may have limited applicability concerning the NCCT based rt-PA treatment of late-presenting patients. Nevertheless, some findings are likely to be transferable. Moreover, Deng et al. (2022), investigated the utility of a NIHSS-ASPECTS-Mismatch (NIHSS score ≥ 8 and ASPECTS ≥ 8) for detecting ischemic penumbra and guiding IVT treatment decisions. The authors observed a good functional outcome without an increasing risk of sICH or mortality in treated patients with this clinical-imaging mismatch. Though it concerned the early timeframe, it is conceivable that this concept is also applicable for late time-windows, since similar strategies are used for therapy decision-making in real world practice (Deng et al., 2022).

Moreover, a meta-analysis of large IVT trials underlines that the beneficial treatment effect of rt-PA may extend the 4.5 h time-window in some patients (Emberson et al., 2014). Further investigations are still needed, but data from WUS-patients underline that the denial of IVT to ischemic stroke patients presenting within the 4.5–6 h time-window and in the absence of advanced imaging techniques seems not reasonable.

Owing to rather rare data concerning thrombolytic therapy in the late time-window based on NCCT, it should be mentioned that there is increasing evidence for mechanical thrombectomy in patients with large vessel occlusions using ASPECTS only (Nagel et al., 2019; Bouslama et al., 2021). As an example, findings of the current CLEAR trial indicate that those patients had comparable clinical and safety outcomes with patients selected by advanced imaging methods (Nguyen et al., 2022).

## What we know and what we need to learn?

As shown above, data on NCCT-based IVT in ischemic stroke patients presenting in the extended time-window is scarce. But what can we transfer from available data and which lessons do we still have to learn about NCCT-based IVT in these patients?

First, do we want to exclude patients from IVT as a consequence of lacking multimodal imaging? Several studies showed that even NCCT is sufficiently safe as an indicator for IVT especially in patients presenting shortly after 4.5 h. Data from WUS/SUO indicate that the sICH risk is not increased when early ischemic changes are not distinct as indicated by an ASPECTS score higher than 7 points. So why should there be an increased risk of IVT-related bleeding if experienced stroke physicians take NCCT and the time-window from symptom onset into account?

Second, multimodal imaging is primarily used to identify patients in the extended time-window who benefit the most from recanalizing strategies, such as IVT (Albers, 2018). The exclusion criteria of ECASS-IV, EXTEND, and EPITHET is implied for patients with a pre-stroke modified Rankin Scale of > 1, different premedication, and a PWI/DWI ratio ≥ 1.2, as well as minimum and maximum PWI and DWI volumes (Davis et al., 2008; Ma et al., 2019; Ringleb et al., 2019). This strict preselection aims to include only those patients who would highly likely benefit from IVT. But only a small percentage of stroke patients fulfill these trial criteria in routine care. Of course, the efficacy of IVT may be limited in patients without beneficial penumbra to ischemic core mismatch, but the higher percentage of patients presenting in the 4.5–6 h time-window suitable for IVT based on NCCT is highly likely to outweigh this aspect. Especially patients with clinical-imaging mismatch—i.e., severe neurological deficits with higher NIHSS and normal NCCT on admission—may benefit from IVT either alone or as bridging therapy to endovascular treatment in the case of large vessel occlusion.

## Conclusion

Although there is limited evidence for NCCT-based thrombolysis in late presenting strokes or strokes of unknown onset, current data suggests that IVT-treatment is safe and results in good functional outcome, especially for WUS and patients presenting within 6 h. Especially in the presence of high ASPECTS and a clinical mismatch between severity of symptoms and radiological findings, the decision should tend toward therapy regardless of availability of advanced imaging. Other advantages using NCCT, beside its almost global availability, is preventing further treatment delay and lower costs as well as fewer radiation exposures and contrast load. Nevertheless, further research is needed in the field of late time-windows as well as larger sample sizes for both, extended and unknown time-window, to verify the non-inferiority of NCCT to advanced imaging in terms of efficacy and safety, especially considering symptomatic ICH.

Finally, NCCT imaging as a more pragmatic alternative to CTP or MRI has the potential to widen the indication for treating patients in the extended or unknown window in case of absence of these imaging techniques. Therefore, patients presenting within 4.5–6 h after symptom onset should not be excluded of IVT due to the absence of advanced neuroimaging if a clinical-imaging mismatch is present.

## Author contributions

JE, RB, and NL provided substantial contributions for literature search and interpretation of data. MJ and SG contributed to the concept and design of the current work. All authors drafted the work, revised it critically for important intellectual content, and provided approval for publication of the content.

## Conflict of interest

The authors declare that the research was conducted in the absence of any commercial or financial relationships that could be construed as a potential conflict of interest.

## Publisher's note

All claims expressed in this article are solely those of the authors and do not necessarily represent those of their affiliated organizations, or those of the publisher, the editors and the reviewers. Any product that may be evaluated in this article, or claim that may be made by its manufacturer, is not guaranteed or endorsed by the publisher.
